# β-blockers after acute myocardial infarction in patients with chronic obstructive pulmonary disease: A nationwide population-based observational study

**DOI:** 10.1371/journal.pone.0213187

**Published:** 2019-03-05

**Authors:** Tse-Hsuan Su, Shang-Hung Chang, Chang-Fu Kuo, Pi-Hua Liu, Yi-Ling Chan

**Affiliations:** 1 Departments of Emergency Medicine, Chang Gung Memorial Hospital Linkou, Taoyuan, Taiwan; 2 Departments of Cardiology, Chang Gung Memorial Hospital Linkou, Taoyuan, Taiwan; 3 Departments of Rheumatology, Chang Gung Memorial Hospital Linkou, Taoyuan, Taiwan; 4 Clinical Informatics and Medical Statistics Research Center, Chang Gung University, Taoyuan, Taiwan; Azienda Ospedaliero Universitaria Careggi, ITALY

## Abstract

**Background:**

Patients with chronic obstructive pulmonary disease (COPD) less often receive β-blockers after acute myocardial infarction (AMI). This may influence their outcomes after AMI. This study evaluated the efficacy of β-blockers after AMI in patients with COPD, compared with non-dihydropyridine calcium channel blockers (NDCCBs) and absence of these two kinds of treatment.

**Methods and results:**

We conducted a nationwide population-based cohort study using data retrieved from Taiwan National Health Insurance Research Database. We collected 28,097 patients with COPD who were hospitalized for AMI between January 2004 and December 2013. After hospital discharge, 24,056 patients returned to outpatient clinics within 14 days (the exposure window). Those who received both β-blockers and NDCCBs (n = 302) were excluded, leaving 23,754 patients for analysis. Patients were classified into the β-blocker group (n = 10,638, 44.8%), the NDCCB group, (n = 1,747, 7.4%) and the control group (n = 11,369, 47.9%) based on their outpatient prescription within the exposure window. The β-blockers group of patients had lower overall mortality risks (adjusted hazard ratio [95% confidence interval]: 0.91 [0.83–0.99] versus the NDCCB group; 0.88 [0.84–0.93] versus the control group), but the risk of major adverse cardiac events within 1 year was not statistically different. β-blockers decreased risks of re-hospitalization for COPD and other respiratory diseases by 12–32%.

**Conclusions:**

The use of β-blockers after AMI was associated with a reduced mortality risk in patients with COPD. β-blockers did not increase the risk of COPD exacerbations.

## Introduction

The mortality rate of acute myocardial infarction (AMI) is higher in patients with chronic obstructive pulmonary disease (COPD) [1]. We have previously reported that patients with COPD had a higher in-hospital, 90-day, and 1-year mortality after AMI in Taiwan [1]. We attributed the higher in-hospital mortality to the under-treatment of these patients during hospitalization. Meanwhile, the long-term outcome of these patients would have been affected more by the outpatient treatment received. β-blockers are less often prescribed to patients with COPD, during and after AMI [2, 3]. The major concern has been the induction of bronchospasm, and some clinicians choose to prescribe non-dihydropyridine calcium channel blockers (NDCCBs) to achieve similar clinical effects, i.e. a slower heart rate and a lower blood pressure [4]. However, evidence supporting the liberal replacement of β-blockers with NDCCBs is lacking. We thus investigated the efficacy of β-blockers in patients with COPD after AMI. Our hypothesis was that β-blockers were associated with an improved survival after AMI in patients with COPD, outperforming both NDCCBs and absence of these two kinds of treatment.

## Materials and methods

### Study populations and data collection

We conducted a retrospective cohort study by using Taiwan National Health Insurance Research Database (NHIRD). Taiwan NHIRD is a large claims database provided by Taiwan National Health Insurance, which is the single-payer, universal, and compulsory healthcare program that covers >99% of Taiwan residents. Taiwan NHIRD contains complete individual diagnosis (both outpatients and inpatients), claimed procedures, and prescriptions. The data were de-identified for research purposes. The study was approved by the Chang Gung Medical Foundation Institutional Review Board, waiving the need for informed consent.

From Taiwan NHIRD, we identified patients with COPD who were hospitalized with the diagnosis of AMI between January 2004 and December 2013. The diagnosis of COPD was confirmed by (1) the presence of the diagnostic codes (International Classification of Diseases, Ninth Revision, Clinical Modification [ICD-9-CM] codes 491, 492, and 496) in the medical record, and (2) the patient had ≥2 outpatient visits or ≥1 hospitalization for COPD during the study period. Patients were identified as having the index AMI only if they received coronary angiography, percutaneous coronary intervention (PCI), or coronary artery bypass graft (CABG) surgery during hospitalization, along with the presence of AMI (ICD-9-CM code 410.x) in discharge diagnoses.

We included patients who were aged >20 years during the indexed AMI hospitalization. We excluded patients who survived AMI but were hospitalized for less than 2 days. For patients with multiple admissions, we included only the first admission.

To circumvent the immortal time bias, we used the landmark analysis in the study [5]. Given patients were usually followed in the outpatient clinic within 2 weeks post-hospital discharge [6], we used the post-discharge Day 14 as the landmark time. Exposure was only evaluated between the discharge date and the landmark time (referred to as the ‘exposure window’). We classified patients into 3 groups according to the outpatient prescriptions in the exposure window: patients who received β-blockers (the β-blocker group), those who received NDCCBs (the NDCCB group), and those who received neither of the two kinds of treatment (the control group). We excluded patients who simultaneously received β-blockers and NDCCBs from analysis.

### Study variables and outcome measurement

We retrieved data from both inpatient and outpatient databases from 1 year prior to the index AMI. We recorded age, sex, socioeconomic status, and associated comorbidities including hypertension, diabetes mellitus, previous AMI, ischemic heart disease, heart failure, stroke, dyslipidemia, chronic kidney disease, and atrial fibrillation. The Charlson Comorbidity Index [7] for individual patients was calculated according to the record of the index AMI hospitalization. We also recorded the inpatient prescriptions and the occurrence of complications of AMI during the index hospitalization, including respiratory failure and shock. Respiratory failure was defined as the use of mechanical ventilatory support. Shock was defined as the use of norepinephrine, dopamine, epinephrine, or intra-aortic balloon pumping. Because Taiwan NHIRD does not contain clinical history or laboratory or examination results that reflect COPD severity (e.g., dyspnea scales or pulmonary function tests data), we alternatively defined the presence of severe COPD if the patient were hospitalized for acute exacerbation within 12 months prior to the index AMI.

The primary outcomes were the 1-year mortality, and the overall mortality till the end of 2013 or the date of patient death, whichever came first. The secondary outcomes were major adverse cardiac events (MACE) and the number of use of acute medical services for COPD or other respiratory diseases within 1 year. MACE included death, repeated revascularization, repeated AMI, and ischemic stroke. Use of acute medical services includes emergency department admissions and hospitalizations.

### Statistical analysis

Patient characteristics, including medical history, comorbidities, in-hospital treatments, and outpatient prescriptions in the exposure window were compared between groups. Categorical data were reported as frequencies (percentages). Numerical data were reported as medians (interquartile ranges). We employed the inverse probability of treatment-weighted (IPTW) approach to balance the intergroup differences in baseline characteristics [8, 9]. IPTW is a weighing methodology that applies to individual subjects, and is also suitable for adjusting groups receiving several treatment alternatives [10]. The weights were derived to estimate the average treatment effects of a representing population [11]. We used generalized boosted models based on 10,000 regression trees to calculate the propensity score of each patient [12], thereby determining the individual weight of each patient. Variables in the propensity score model included age, sex, socioeconomic status, hospital length of stay (LOS) for the index AMI, comorbidities, previous outpatient treatment for COPD, inpatient treatments, complications of AMI during hospitalization, and other outpatient prescriptions in the exposure window. After applying the weight to individual patients, the variables in the propensity score model have been adjusted between groups. The characteristics of different treatment groups in the propensity score model were then similar and balanced, allowing us to compare the estimated treatment effects between groups. To confirm the balancing between groups, we calculated the absolute standardized mean differences between groups for every baseline covariate. A threshold of <0.1 indicated well balancing [13].

We evaluated all-cause mortality and MACE-related outcomes within 1-year using weighted Kaplan-Meier analysis. We used multivariable Cox proportional hazard models to calculated crude and adjusted weighted hazard ratios (HRs), and assessed the proportional assumption graphically. Regarding the number of use of acute medical services within 1 year, we used negative binomial models to calculate the incidence rate ratio. The incidence rate was defined as the number of use of acute medical services divided by person-year. The prior-year number of use of acute medical services for COPD and other respiratory diseases were also used for adjustment. In both the survival analysis and the negative binomial models, we also adjusted the same variables (S1 Table). A two-tailed *p-value* <0.05 indicated statistical significance. Analyses were performed in SAS version 9.4 (SAS Institute, Inc.).

### Sensitivity analyses and subgroup analyses

We conducted several sensitivity analyses to test the robustness of the results from different perspectives. Firstly, we included only patients receiving PCI or CABG in the index hospitalization, so as to eliminate the confounding from the false-positive diagnosis of AMI. Secondly, we analyzed patients with the diagnosis of COPD prior to the index AMI, so that patients who were falsely diagnosed as having COPD during the index AMI hospitalization would not dilute the harmful effect of β-blockers. Thirdly, we analyzed patients who did not receive β-blockers or NDCCBs within 6 months prior to the index AMI. By doing so we employed the incident-user design to reduce the confounding from previous therapies [14]. Finally, we changed the landmark time to post-discharge Day 28 to find out whether a longer exposure window would still generate similar results.

We also conducted subgroup analyses to look into conditions in which the use of β-blockers or NDCCBs might have been hampered or facilitated: (1) severe COPD or respiratory failure; and (2) presence of congested heart failure or shock during admissions. Congested heart failure or shock was defined by the use of diuretics, norepinephrine, dopamine, epinephrine, or intra-aortic balloon pumping. We also compared mortality and cardiovascular outcomes in patients who received or did not receive PCI or CABG during the index MI. For all sensitivity and the subgroup analyses, we used the same IPTW approach and adjusted for the same covariates.

## Results

### Baseline characteristics

**Fig 1:**
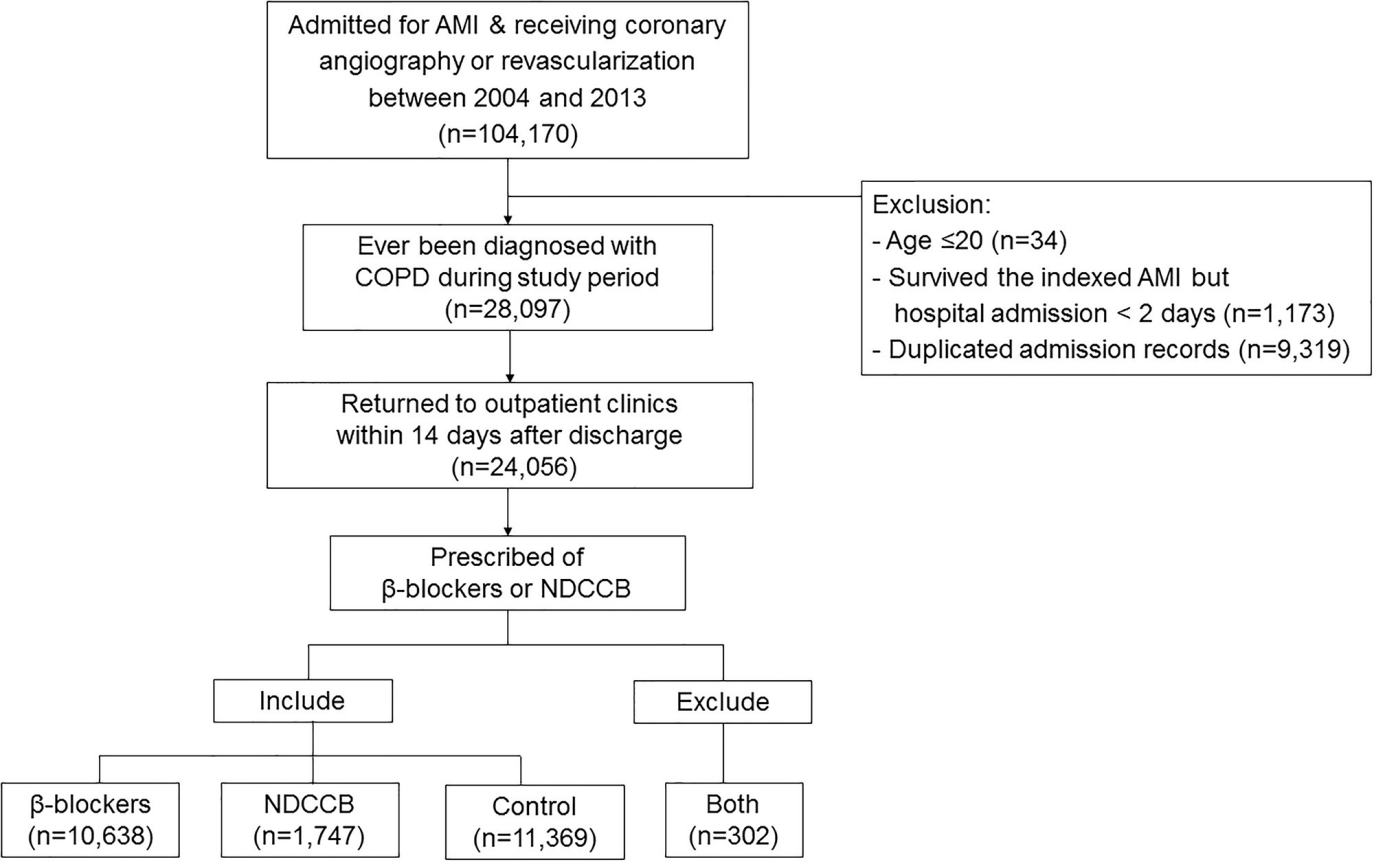
Patient inclusion and exclusion. Abbreviations: AMI, acute myocardial infarction; COPD, chronic obstructive pulmonary disease; NDCCB, non-dihydropyridine calcium channel blockers.

From Taiwan NHIRD, we identified 104,170 patients who were hospitalized during the study period with the diagnosis of AMI. Among them, 28,097 had COPD, in which 24,056 were followed in the outpatient clinic within 2 weeks after hospital discharge. After excluding patients who simultaneously received β-blockers and NDCCBs (n = 302), the rest 23,754 were classified into the β-blocker (n = 10,638; 44.8%), the NDCCB (n = 1,747; 7.4%) and the control groups (n = 11,369; 47.9%) (Fig 1). Among the 10,638 patients of the β-blocker group, 5,136 (48.3%) used cardioselective β-blockers, 5,502 (51.7%) used non-selective β-blockers, and 95 (0.9%) patients used both selective and non-selective β-blockers in the exposure window.

Regarding the baseline characteristics of each group, patients in the β-blocker group were slightly younger (Table 1); they more often had dyslipidemia and diabetic mellitus, but less often had heart failure and severe COPD. During the index AMI hospitalization, the β-blocker group of patients less often received diuretics, dobutamine, anti-arrhythmics, and treatment for COPD (Table 2). They also less often developed respiratory failure, but slightly more often developed shock. To the contrary, the NDCCB group of patients more frequently had severe COPD and received treatment for COPD before, during, and after the index AMI hospitalization. They more often received dobutamine during hospitalization. The proportion of patients who had severe COPD in the control group was between the other groups. However, a higher proportion of these patients developed respiratory failure and shock during the index AMI hospitalization. The follow-up time (mean ± standard deviation) was 3.9 ± 2.7, 3.8 ± 2.8, and 3.5 ± 2.7 years for the β-blocker, the NDCCB and the control groups, respectively.

**Table 1:** Baseline characteristics of patients with chronic obstructive pulmonary disease and acute myocardial infarction.

	Before weighting				After weighting			
Characteristics	β-blockers n = 10638	NDCCB n = 1747	Control n = 11369	MASD*	β-blockers n = 10638	NDCCB n = 1747	Control n = 11369	MASD*
Age, y	70 (61–78)	74 (66–80)	74(65–80)	0.2770	72 (63–79)	72 (64–79)	72 (63–79)	0.0260
Sex: male, %	8157 (77)	1317 (75)	8721 (77)	0.0310	8168 (77)	1359 (78)	8740 (77))	0.0240
Socioeconomic status^†^				0.1110				0.0320
≥40000	884 (8)	95 (5)	606 (5)		710 (7)	101 (6)	754 (7)	
20000–39999	3427 (32)	572 (33)	3893 (34)		3534 (33)	570 (33)	3819 (34)	
1–19999	2967 (28)	525 (30)	3389 (30)		3084 (29)	511 (29)	3291 (29)	
Dependent	3360 (32)	555 (32)	3481 (31)		3310 (31)	565 (32)	3505 (31)	
Hospital Duration, d	7 (5–11)	7 (5–12)	8 (5–14)	0.2000	7 (5–12)	7 (5–11)	7 (5–12)	0.0710
Charlson comorbidity index	2 (1–3)	2 (1–3)	2 (1–4)	0.0970	2 (1–3)	2 (1–3)	2 (1–3)	0.0350
Chronic ischemic heart, %	4233 (40)	769 (44)	4631 (41)	0.0860	4318 (41)	720 (41)	4558 (40)	0.0230
Hypertension, %	7262 (68)	1191 (68)	7442 (65)	0.0600	7158 (67)	1185 (68)	7576 (67)	0.0260
Dyslipidemia, %	3378 (32)	477 (27)	3136 (28)	0.0960	3159 (30)	508 (29)	3307 (29)	0.0130
Congestive heart failure, %	1347 (13)	291 (17)	1941 (17)	0.1300	1551 (15)	246 (14)	1716 (15)	0.0300
Stroke, %	1273 (12)	218 (12)	1690 (15)	0.0890	1393 (13)	212 (12)	1549 (14)	0.0460
Diabetes mellitus, %	3926 (37)	546 (31)	4141 (36)	0.1180	3862 (36)	607 (35)	4131 (36)	0.0330
Chronic kidney disease, %	1079 (10)	152 (9)	1278 (11)	0.0850	1083 (10)	158 (9)	1200 (11)	0.0500
Atrial fibrillation, %	383 (4)	108 (6)	534 (5)	0.1320	452 (4)	70 (4)	473 (4)	0.0130
Severe obstructive lung, %	748 (7)	398 (23)	1586 (14)	0.5440	1143 (11)	215 (12)	1323 (12)	0.0540

Patients were classified into the β-blockers, the non-dihydropyridine calcium channel blocker (NDCCB), and the control groups according to the outpatient prescription within 2 weeks after hospital discharge.

Values are median (IQR) or n (%)

* MASD: maximum absolute standardized mean difference between the groups

^†^ Socioeconomic status: monthly household incomes, in Taiwan Dollars.

**Table 2:** In-hospital treatments and events in patients with chronic obstructive pulmonary disease and acute myocardial infarction.

	Before weighting				After weighting			
No. (%)	β-blockers n = 10638	NDCCB n = 1747	Control n = 11369	MASD*	β-blockers n = 10638	NDCCB n = 1747	Control n = 11369	MASD*
ACEI/ARB	8694 (82)	1195 (68)	8461 (74)	0.3320	8233 (77)	1327 (76)	8740 (77)	0.0360
Nitrate	10211 (96)	1653 (95)	10742 (94)	0.0703	10140 (95)	1667 (95)	10815 (95)	0.0136
Statins	6344 (60)	793 (45)	5654 (50)	0.2880	5730 (54)	914 (52)	6107 (54)	0.0310
Diuretics	5321 (50)	934 (53)	6772 (60)	0.1910	5816 (55)	910 (52)	6272 (55)	0.0610
Digoxin	911 (9)	229 (13)	1521 (13)	0.1670	1175 (11)	175 (10)	1255 (11)	0.0350
Anti-arrhythmics	1739 (16)	332 (19)	2665 (23)	0.1900	2073 (19)	330 (19)	2260 (20)	0.0270
Dobutamine	1592 (15)	579 (33)	2978 (26)	0.4780	2229 (21)	378 (22)	2473 (22)	0.0210
Systemic steroid	2288 (22)	717 (41)	3872 (34)	0.4560	2988 (28)	493 (28)	3319 (29)	0.0260
Inhalational bronchodilators/steroids	2439 (23)	813 (47)	4449 (39)	0.5461	332 (31)	579 (33)	3687 (32)	0.0404
Theophyllin	870 (8)	591 (34)	2288 (20)	0.7950	1597 (15)	294 (17)	1807 (16)	0.0560
PCI	8661 (81)	1098 (63)	8314 (73)	0.4540	8191 (77)	1316 (75)	8641 (76)	0.0410
CABG	505 (5)	105 (6)	1042 (9)	0.2040	719 (7)	110 (6)	803 (7)	0.0360
Respiratory failure	1531 (14)	291 (17)	2371 (21)	0.1820	1859 (17)	270 (15)	2014 (18)	0.0640
Shock	3047 (29)	480 (27)	4225 (37)	0.2150	3471 (33)	534 (31)	3742 (33)	0.0510

Patients were classified into the β-blockers, the NDCCB, and the control groups according to the outpatient prescription within 2 weeks after hospital discharge.

Abbreviations: ACEI/ARB: angiotensin converting enzyme inhibitors/angiotensin II receptor blockers; CABG: coronary artery bypass grafting; NDCCB: non- dihydropyridine calcium channel blockers; PCI, percutaneous coronary intervention.

* MASD, maximum absolute standardized mean difference between the groups

After applying the IPTW methodology, the baseline characteristics (Table 1), in-hospital treatments (Table 2), and outpatient prescriptions in the exposure window (S2 Table) became well balanced between groups. The maximum standardized mean differences between groups were all <0.1.

### Outcomes

**Fig 2:**
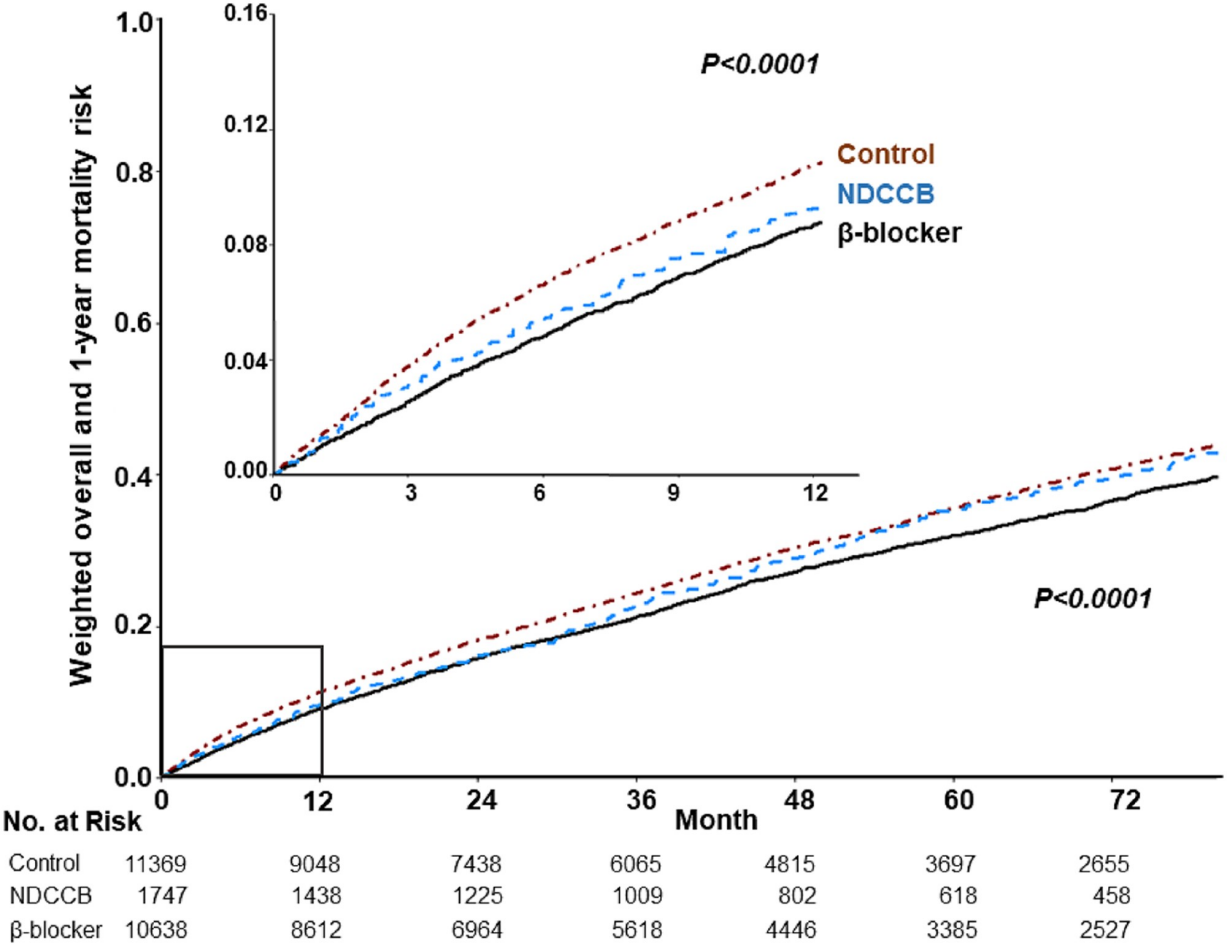
Cumulative risk curves of 1-year and overall mortality. Abbreviations: NDCCB, non-dihydropyridine calcium channel blockers.

**Fig 3:**
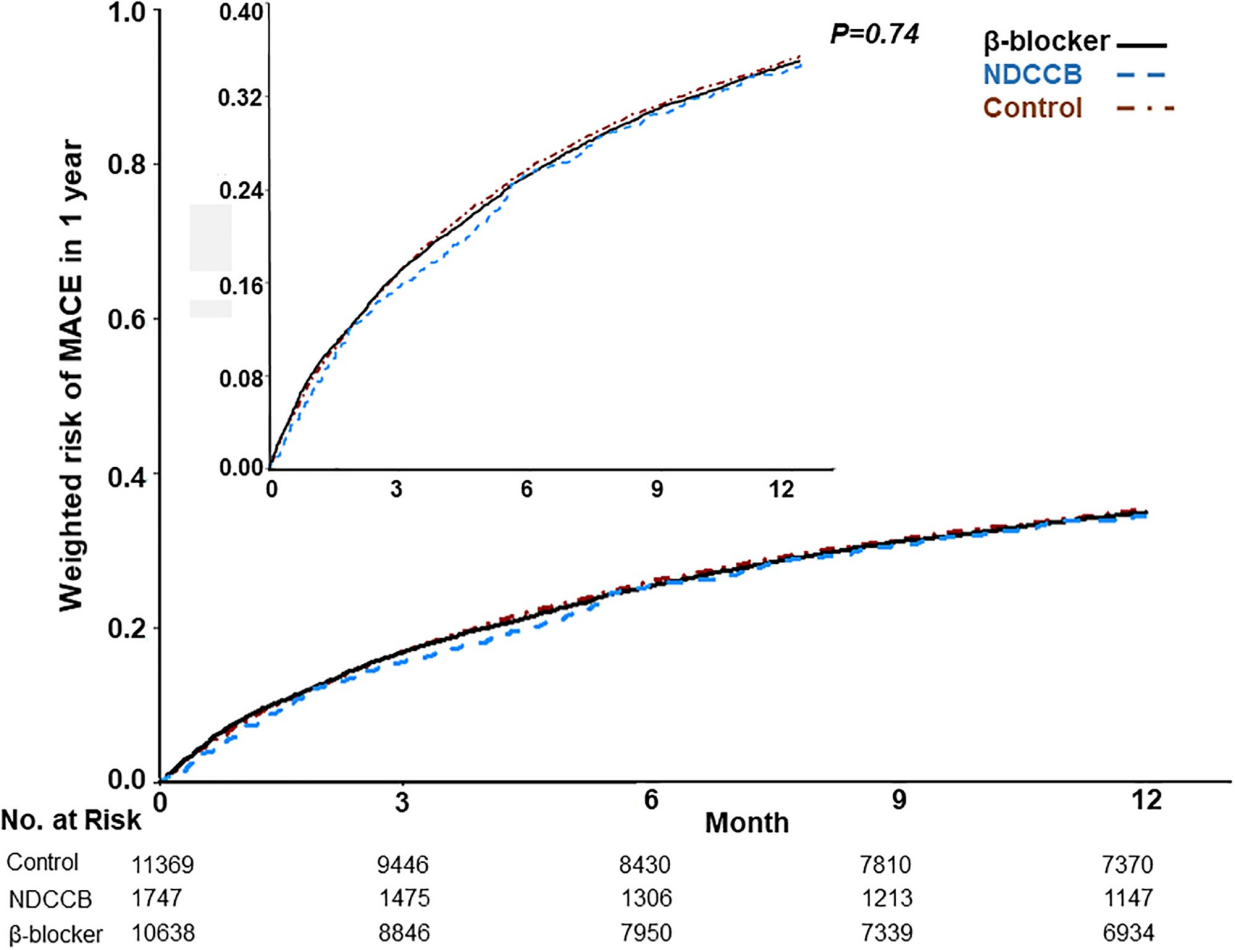
Cumulative risk curves of major adverse cardiac events (MACE) in 1 year. Abbreviations: NDCCB, non-dihydropyridine calcium channel blockers.

Regarding the 1-year and the overall mortality, the weighted Kaplan-Meier plots showed significant differences between the three groups of patients (*p* <0.0001, log-rank test, Fig 2). However, we observed no differences in the occurrence of MACE within 1 year between groups (*p* = 0.74, Fig 3), both before and after weighting. The β-blocker group of patients showed a lower 1-year and overall mortality risk compared with the control group (Table 2), and a lower overall mortality risk compared with the NDCCB group. There was a trend that the β-blocker group of patients had higher risks of repeated AMI and revascularization (Table 3). However, compared with the NDCCB and the control group, the β-blocker group of patients less often used acute medical services for COPD or other respiratory diseases.

**Table 3:** Outcomes of patients with chronic obstructive pulmonary disease and acute myocardial infarction.

	Weighted incidence rate (per 100 person-years)			Adjusted weighted rate ratio	
	β-blockers	NDCCB	Control	β-blockers vs. Control	β-blockers vs. NDCCB
1-year mortality	9.2	9.9	11.6	0.81 (0.74–0.88)***	0.90 (0.77–1.07)
Overall mortality	8.0	8.6	9.2	0.88 (0.84–0.93)***	0.91 (0.83–0.99)*
MACE in 1 year	45.7	44.7	46.4	0.98 (0.94–1.03)	1.01 (0.93–1.10)
Repeated MI	10.4	9.6	8.7	1.08 (0.99, 1.18)	1.17 (0.98, 1.40)
Repeated revascularization	28.1	26.0	26.6	1.04 (0.98, 1.10)	1.05 (0.95, 1.17)
Ischemic stroke	3.7	3.7	3.8	0.98 (0.85, 1.13)	0.97 (0.74, 1.26)
Acute medical services use in 1 year^†^					
Obstructive lung disease	27.9	39.9	36.6	0.74 (0.69–0.80)***	0.68 (0.60–0.78)***
Respiratory diseases	68.1	77.1	76.6	0.88 (0.84–0.93)***	0.88 (0.80–0.97)*

*p <0.05,

**p <0.01,

***p <0.001.

^†^ Incidence rate per 10 person-years

Patients were classified into the β-blockers, the non-dihydropyridine calcium channel blocker (NDCCB), and the control groups according to the outpatient prescription within 2 weeks after hospital discharge.

Abbreviations: MACE, major adverse cardiac events;

### Sensitivity and subgroup analyses

All sensitivity analyses yielded results that were consistent with the original analysis, namely the β-blocker group of patients had a lower overall mortality risk, and less often used acute medical services for COPD and other respiratory diseases compared with the NDCCB and the control groups of patients (S1 Fig). However, new users of β-blocker did not show a lower mortality risk compared with new users of NDCCBs.

In the subgroup analysis, β-blockers similarly posed a lower 1-year and overall mortality risk. For patients with congested heart failure or shock, there seemed a trend towards a lower overall mortality risk for the β-blocker group of patients than the NDCCB group of patients (*p* = 0.064). β-blockers also offered more protection than NDCCBs from using acute medical services for COPD and other respiratory diseases, especially for patients with severe COPD, and those who had developed respiratory failure, congested heart failure or shock during the index AMI hospitalization (S2 Fig).

Most patients received revascularization during the index MI (n = 19,528, 82.2%). In these patients, the β-blocker group of patients had lower rate ratios of 1-year and overall mortality. To the contrary, in patients who did not receive revascularization during indexed MI (n = 4,226, 17.8%), the NDCCB group of patients had a significantly lower rate ratio of repeated MI within 1 year (S3 Table).

## Discussion

### Principal findings

In this nationwide population-based study, we demonstrated that patients with COPD and AMI who received β-blockers within two weeks after hospital discharge had a 9% and 12% risk reduction in overall mortality, compared respectively with those receiving NDCCBs or neither of these two kinds of treatment. β-blockers were associated with lower risks of using acute medical services for COPD and other respiratory diseases. This protective effect was even greater in patients with more severe COPD or AMI. However, no differences were present regarding the risk of MACE within 1 year between treatment groups.

### Compare with previous studies

Previous studies demonstrated that β-blockers improved the survival after AMI in patients with COPD [1, 2, 15]. However, these studies only compared β-blocker users with non-users, not like our study which compared patients receiving β-blockers, NDCCBs, and absence of these two kinds of treatment. We also specifically addressed patients with severe COPD and AMI. Contrary to the general perception that β-blockers may deteriorate lung function, several studies as well as our data revealed that β-blockers actually reduced risks of acute exacerbation, hospitalization, and mortality [16–19]. Andell et al. demonstrated that in the SWEDEHEART registry between 2005 and 2010, patients with COPD and AMI had a lower risk for overall mortality if β-blockers were included in the discharge medications (adjusted HR, 0.87; 95% CI, 0.87–0.98) [20]. However, the 1-year mortality risk was not different. Quint et al. found that in the British national registry of AMI between 2003 and 2008, patients with COPD who received β-blockers had a lower risk of mortality (adjusted HR, 0.72; 95% CI, 0.57–0.90) [21]. These two studies did not specify how many of the β-blocker non-users had received NDCCBs.

In patients who received revascularization during the index MI, the risks of developing MACE within 1 year were similar between treatment groups. However, in patients who did not receive revascularization during the index MI, the risks of developing repeated MI were lower in the NDCCB group compared with the β-blocker group, while no difference was observed between the β-blocker group and the control group of patients. We speculated that in patients who did not receive revascularization during the index MI, there might have been more patients who actually had coronary vasospasm, thus having less repeated MI after receiving NDCCBs. Unfortunately we cannot confirm this speculation since we could not obtain the coronary catheterization report and document the presence of coronary vasospasm in these patients. The lack of survival benefit of β-blockers in patients not receiving revascularization during the index MI might be attributable to the comorbidities, given the incidence rates of 1-year and overall mortality were all higher in all treatment groups.

We wondered whether NDCCBs could be equally protective as β-blockers for patients with COPD and AMI. Theoretically NDCCBs are a safer choice for patients with reactive airway diseases as they do not induce bronchospasm. Current AMI treatment guidelines also recommend the use of NDCCBs if β-blockers are contraindicated or intolerable for the patient. However, evidence supporting the liberal replacement of β-blockers with NDCCBs is lacking, and this practice is not without its concern. For example, NDCCBs are deemed unsuitable for patients with systolic heart failure [22–24], not like β-blockers which can improve their survival with careful titration [24, 25]. We particularly addressed the number of use of acute medical services in our patients, to see if the NDCCB group of patients had less frequent acute exacerbation of COPD. Unexpectedly, the result ended up toward the opposite direction. That is, in our cohort β-blockers exhibited greater efficacy in reducing mortality risks and the frequency of COPD exacerbation than NDCCBs, which was even greater for patients with more severe COPD or AMI. In other words, NDCCBs were simply not safer than β-blockers for patients with COPD and AMI. We thus urge that NDCCBs should be prescribed not for other uncorroborated concerns, but only if the patient is actually contraindicated or intolerable to β-blockers.

Dong et al. [26] pooled data from several claims databases and demonstrated a protective effect of cardioselective β-blockers over NDCCBs in preventing COPD hospitalizations within as early as 30 days. However, the authors attributed this effect to a potential bias, arguing that the cardioselective β-blockers could not have possibly exerted its protective effects within only 30 days. We think this conclusion is rather assertive, because we do not see why the protective effect of cardioselective β-blockers surely cannot appear within 30 days. Moreover, we suspected the use of COPD admission within 30 days after the use of cardioselective β-blockers can hardly serve as a negative control, as the decision on whether to admit a patient with COPD could be quite different between countries. In fact, the incidence of COPD hospitalization in their cohort was quite diverse between databases, with that of Taiwan patients only half of that of US and Italian patients. Hence, to the contrary of Dong et al., we believe that β-blockers exert a protective effect on patients with COPD and AMI.

Our results showed that patients received β-blockers were associated with decreased risk of rehospitalization for COPD and other respiratory diseases. Possible explanations could be in patients with COPD, the use of beta-blockers does not seem to decline the forced expiratory volume in one second [27]. Some studies even showed that beta-blockers decrease the risk of acute exacerbation of COPD [28–30]. Animal studies reported that administration of beta-blockers increased the density of pulmonary beta-receptors, which in turn decreased the bronchoconstriction attributable to their overexpression. [31, 32] Besides, arrhythmia and tachycardia were also considered triggers for the acute exacerbations of COPD. The use of beta-blockers could reduce tachycardia and alleviate arrhythmia.[33]

The rationale for using β-blockers or NDCCBs in patients with AMI is to decrease myocardial oxygen demand, through reducing left ventricular afterload and slowing the heart rate. However, β-blockers seem capable of exerting additional beneficial effects on patients with COPD. Several mechanisms may contribute to this extra protection, including the up-regulation of β_2_ adrenergic receptors in the lungs, the reduction of sympathetic tone, the inhibition of cardiac stimulation by catecholamines or β-agonists, the modulation of chronic inflammation, the increased production of nitric oxide in vascular smooth muscle, and the decrease in alveolar-capillary membrane diffusion capacity [34, 35]. Importantly, COPD itself poses an increased burden to the cardiovascular system, possibly through increasing arterial stiffness and inducing systemic inflammation [36]. Although diltiazem also appears to exert anti-inflammatory and sympatholytic effects [37, 38] its protective effect was somehow not as prominent in our patient cohort.

### Strengths and limitations

To our knowledge, this is the first nationwide, population-based study to compare the treatment effect of β-blockers, NDCCBs, and absence of these two kinds of treatment after AMI in patients with COPD. Our study used the landmark analysis method to circumvent the immortal time bias. We also used the IPTW methodology to adequately balance the differences between each treatment groups.

Nevertheless, our study has its limitations. First, we extracted our data from the claims database, which did not provide personal history, laboratory, and examination results. Thus we were unable to obtain relevant information such as body mass index or the status of tobacco use, as well as examination reports like left ventricular ejection fractions and pulmonary function test results. We therefore had to assume the patient’s clinical conditions from the treatmeplusnt they received, e.g. heart failure by receiving dobutamine or diuretics, and more severe COPD if the patient was hospitalized. These surrogate markers of disease severity could be inaccurate. Nevertheless, we still deemed our results reliable as the results from our sensitivity and subgroup analyses all went in the same direction. Second, the diagnosis of COPD was based on diagnostic codes, which again might not be totally accurate [39]. Moreover, it may be difficult to diagnose COPD and to grade its severity in patients with AMI, for spirometry results can be misleading, especially in patients with heart failure [40, 41]. We addressed this potential shortcomings in our sensitivity analyses, and their results were consistent with the original model. Third, as an observational study, our results could inherently be biased by the unmeasurable and unrecorded confounding factors (e.g., confounding by indications). Therefore, we still need a randomized controlled trial to unbiasedly confirm the beneficial effect of β-blockers in patients with COPD and AMI.

## Conclusions

The use of β-blockers was associated with a lower mortality rate in patients with COPD after AMI. β-blockers were not associated with an increased risk of hospitalization for COPD or respiratory diseases, even in patients with severe COPD. However, the use of β-blockers did not decrease the occurrence of MACE within 1 year after MI in our patient cohort.

## Supporting information

S1 Fig Sensitivity analyses of the effects of β-blockers and non-dihydropyridine calcium channel blockers in patients with chronic obstructive pulmonary disease and acute myocardial infarction. Abbreviations: AMI, acute myocardial infarction; CABG, coronary artery bypass graft; CI, confidence interval; COPD, chronic obstructive pulmonary disease; NDCCB, non-dihydropyridine calcium channel blockers; PCI, primary cutaneous intervention; RR, relative risks; SA, sensitivity analysis.
(TIF)

S2 Fig Subgroup analyses of the effects of β-blockers and non-dihydropyridine calcium channel blockers in patients with chronic obstructive pulmonary disease and acute myocardial infarction. *, interaction terms between subgroups were significant. The subgroups ‘Obstructive Lung Diseases’ and ‘Respiratory Diseases’ indicated the number of acute medical services uses for COPD and other respiratory diseases, respectively. Abbreviations: CI, confidence interval; COPD, chronic obstructive pulmonary disease; NDCCB, non-dihydropyridine calcium channel blockers; RR, relative risks.
(PDF)

S1 Table Variables adjusted in the survival and negative binomial models. (DOCX)

S2 Table Outpatient treatment in patients with chronic obstructive pulmonary disease and acute myocardial infarction. (DOCX)

S3 Table Mortality and cardiovascular outcomes in patients received revascularization or not during the acute myocardial infarction. (DOCX)
