# Supplementary figures and images for: β-blockers after acute myocardial infarction in patients with chronic obstructive pulmonary disease: A nationwide population-based observational study
Source: PLoS One. 2019 Mar 5;14(3):e0213187. doi: 10.1371/journal.pone.0213187 (PMC6400336; doi:10.1371/journal.pone.0213187)

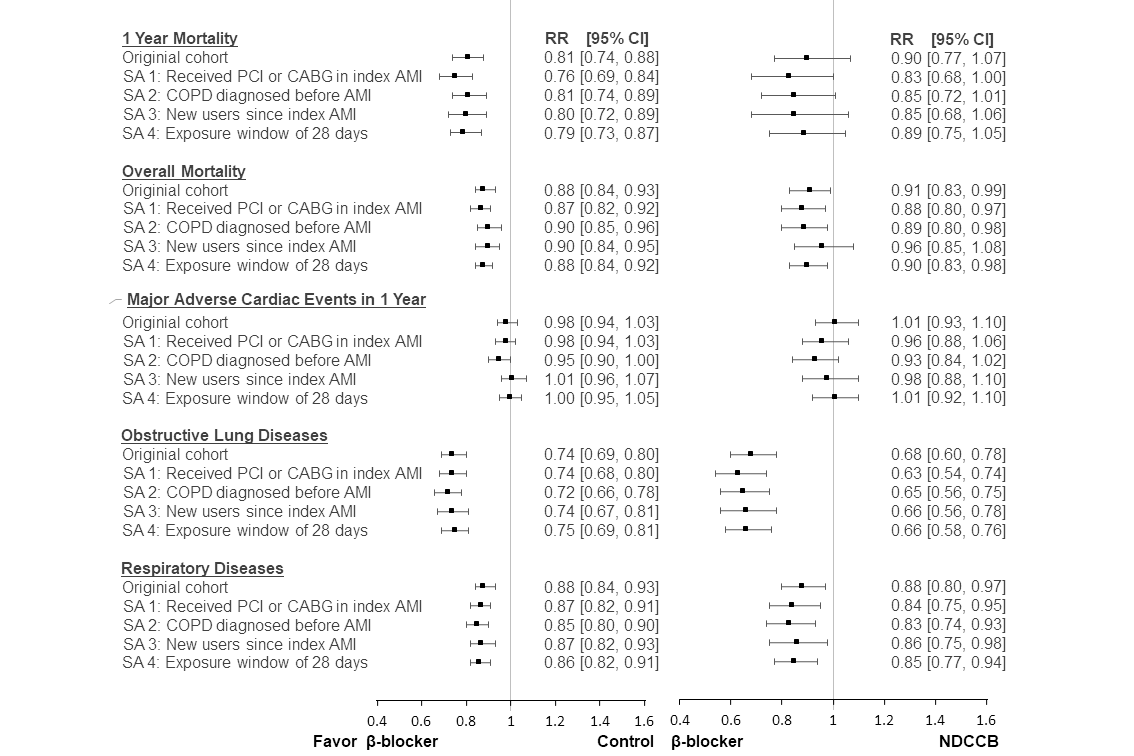

Supplement: S1 Fig — Abbreviations: AMI, acute myocardial infarction; CABG, coronary artery bypass graft; CI, confidence interval; COPD, chronic obstructive pulmonary disease; NDCCB, non-dihydropyridine calcium channel blockers; PCI, primary cutaneous intervention; RR, relative risks; SA, sensitivity analysis. (TIF) [file pone.0213187.s001.tif]
