# Supplementary material for: β-blockers after acute myocardial infarction in patients with chronic obstructive pulmonary disease: A nationwide population-based observational study
Source: PLoS One. 2019 Mar 5;14(3):e0213187. doi: 10.1371/journal.pone.0213187 (PMC6400336; doi:10.1371/journal.pone.0213187)
Supplement: S2 Fig — *, interaction terms between subgroups were significant. The subgroups ‘Obstructive Lung Diseases’ and ‘Respiratory Diseases’ indicated the number of acute medical services uses for COPD and other respiratory diseases, respectively. Abbreviations: CI, confidence interval; COPD, chronic obstructive pulmonary disease; NDCCB, non-dihydropyridine calcium channel blockers; RR, relative risks. (PDF) [file pone.0213187.s002.pdf]

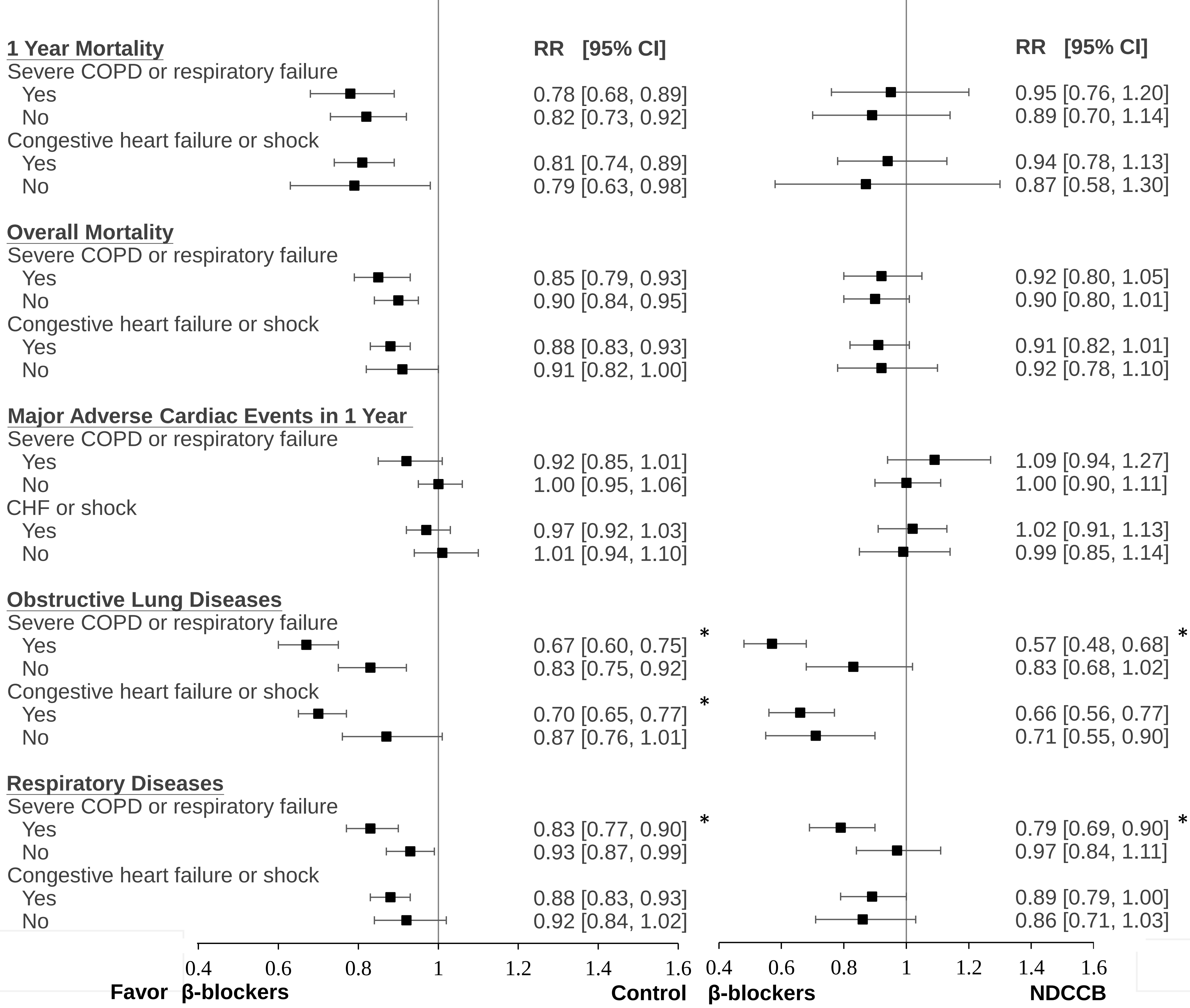

1 Year Mortality
RR [95% CI]
RR [95% CI]
Severe COPD or respiratory failure
Yes 0.78 [0.68, 0.89] 0.95 [0.76, 1.20]
No 0.82 [0.73, 0.92] 0.89 [0.70, 1.14]
Congestive heart failure or shock
Yes 0.81 [0.74, 0.89] 0.94 [0.78, 1.13]
No 0.79 [0.63, 0.98] 0.87 [0.58, 1.30]
Overall Mortality
Severe COPD or respiratory failure
Yes 0.85 [0.79, 0.93] 0.92 [0.80, 1.05]
No 0.90 [0.84, 0.95] 0.90 [0.80, 1.01]
Congestive heart failure or shock
Yes 0.88 [0.83, 0.93] 0.91 [0.82, 1.01]
No 0.91 [0.82, 1.00] 0.92 [0.78, 1.10]
Major Adverse Cardiac Events in 1 Year
Severe COPD or respiratory failure
Yes 0.92 [0.85, 1.01] 1.09 [0.94, 1.27]
No 1.00 [0.95, 1.06] 1.00 [0.90, 1.11]
CHF or shock
Yes 0.97 [0.92, 1.03] 1.02 [0.91, 1.13]
No 1.01 [0.94, 1.10] 0.99 [0.85, 1.14]
Obstructive Lung Diseases
Severe COPD or respiratory failure
Yes 0.67 [0.60, 0.75] * 0.57 [0.48, 0.68] *
No 0.83 [0.75, 0.92] 0.83 [0.68, 1.02]
Congestive heart failure or shock
Yes 0.70 [0.65, 0.77] * 0.66 [0.56, 0.77]
No 0.87 [0.76, 1.01] 0.71 [0.55, 0.90]
Respiratory Diseases
Severe COPD or respiratory failure
Yes 0.83 [0.77, 0.90] * 0.79 [0.69, 0.90] *
No 0.93 [0.87, 0.99] 0.97 [0.84, 1.11]
Congestive heart failure or shock
Yes 0.88 [0.83, 0.93] 0.89 [0.79, 1.00]
No 0.92 [0.84, 1.02] 0.86 [0.71, 1.03]
0.4 0.6 0.8 1 1.2 1.4 1.6
0.4 0.6 0.8 1 1.2 1.4 1.6
Favor β-blockers Control
β-blockers NDCCB
