# Supplementary material for: β-blockers after acute myocardial infarction in patients with chronic obstructive pulmonary disease: A nationwide population-based observational study
Source: PLoS One. 2019 Mar 5;14(3):e0213187. doi: 10.1371/journal.pone.0213187 (PMC6400336; doi:10.1371/journal.pone.0213187)
Supplement: S1 Table — (DOCX) [file pone.0213187.s003.docx]

**S1 Table Variables adjusted in the survival and negative binomial models**

We adjusted for age, sex, socioeconomic status, hospital length of stay for the index AMI, comorbidities (prior ischemic heart disease, hypertension, dyslipidemia, congestive heart failure, stroke, diabetes, chronic kidney disease, atrial fibrillation, and severe COPD), in-hospital treatment and complications (including the use of dobutamine, systemic steroids, inhalational bronchodilator/steroids, receiving PCI, CABG, or experienced respiratory failure , and shock), and outpatient prescriptions after AMI ( including ACEI/ARB, dihydropyridine calcium channel blockers, nitrates, statins, diuretics, digoxin, anti-arrhythmics, inhalational bronchodilators/steroids, and theophylline).

Abbreviations:

ACEI/ARB, angiotensin converting enzyme inhibitors/angiotensin II receptor blockers;

AMI, acute myocardial infarction; CABG: coronary artery bypass grafting; COPD, chronic obstructive pulmonary disease; PCI, percutaneous coronary intervention;
