# Supplementary material for: β-blockers after acute myocardial infarction in patients with chronic obstructive pulmonary disease: A nationwide population-based observational study
Source: PLoS One. 2019 Mar 5;14(3):e0213187. doi: 10.1371/journal.pone.0213187 (PMC6400336; doi:10.1371/journal.pone.0213187)
Supplement: S2 Table — (DOCX) [file pone.0213187.s004.docx]

**S2 Table Outpatient treatment in patients with chronic obstructive pulmonary disease and acute myocardial infarction.**

|  | **Before weighting** | | | |  | **After weighting** | | | |
| --- | --- | --- | --- | --- | --- | --- | --- | --- | --- |
| No. (%) | β-blockers n=10638 | NDCCB n=1747 | Control  n=11369 | MASD^*^ |  | β-blockers n=10638 | NDCCB n=1747 | Control  n=11369 | MASD^*^ |
| ACEI/ARB | 7211 (68) | 838 (48) | 5960 (52) | 0.4150 |  | 6374 (60) | 1001 (57) | 6703 (59) | 0.0550 |
| Nitrate | 5125 (48) | 931 (53) | 4937 (43) | 0.1970 |  | 5039 (47) | 837 (48) | 5262 (46) | 0.0330 |
| Statins | 5643 (53) | 642 (37) | 4136 (36) | 0.3330 |  | 4768 (45) | 753 (43) | 4970 (44) | 0.0340 |
| Diuretics | 3484 (33) | 601 (34) | 4205 (37) | 0.0900 |  | 3800 (36) | 589 (34) | 4016 (35) | 0.0420 |
| Digoxin | 391 (4) | 92 (5) | 809 (7) | 0.1780 |  | 553 (5) | 81 (5) | 625 (5) | 0.0440 |
| Anti-arrhythmics | 597 (6) | 137 (8) | 1123 (10) | 0.1810 |  | 817 (8) | 132 (8) | 905 (8) | 0.0170 |
| Inhalational bronchodilators/ steroids | 194 (2) | 204 (12) | 594 (5) | 0.5590 |  | 433 (4) | 74 (4) | 479 (4) | 0.0110 |
| Theophyllin | 759 (7) | 513 (29) | 1765 (16) | 0.7320 |  | 1315 (12) | 245 (14) | 1470 (13) | 0.0560 |

Patients were classified into the β-blockers, the non-dihydropyridine calcium channel blocker (NDCCB), and the control groups according to the outpatient prescription within 2 weeks after hospital discharge.

Abbreviations: ACEI/ARB, angiotensin converting enzyme inhibitors/angiotensin II receptor blockers;

*: MASD: maximum absolute standardized mean difference between the groups
