# Supplementary material for: β-blockers after acute myocardial infarction in patients with chronic obstructive pulmonary disease: A nationwide population-based observational study
Source: PLoS One. 2019 Mar 5;14(3):e0213187. doi: 10.1371/journal.pone.0213187 (PMC6400336; doi:10.1371/journal.pone.0213187)
Supplement: S3 Table — (DOCX) [file pone.0213187.s005.docx]

**S3 Table. Mortality and cardiovascular outcomes in patients received revascularization or not during the acute myocardial infarction**

| **Patients receiving PCI/CABG N=19528** | **Weighted incidence rate (per 100 person-years)** | | | **Adjusted weighted rate ratio** | |
| --- | --- | --- | --- | --- | --- |
|  | β-blockers | NDCCB | Control | β-blockers vs. Control | β-blockers vs. NDCCB |
| 1-year mortality | 8.0 | 9.4 | 10.7 | 0.76(0.69-0.84)*** | 0.83(0.68-1.00)* |
| Overall mortality | 7.5 | 8.3 | 8.7 | 0.87(0.82-0.92)*** | 0.88(0.80-0.97)* |
| MACE in 1 year | 46.3 | 48.2 | 47.2 | 0.98(0.94-1.03) | 1.01 (0.93-1.10) |
| Repeated MI | 10.2 | 9.3 | 9.3 | 1.10(1.00-1.21) | 0.96(0.88-1.06) |
| Repeated revascularization | 30.4 | 29.9 | 28.8 | 1.05(0.99-1.11) | 1.02(0.91-1.14) |
| Repeated ischemic stroke | 3.4 | 3.9 | 3.7 | 0.92(0.79-1.08) | 0.85(0.64-1.14) |

**p* <0.05, ***p* <0.01, ****p* <0.001.
Patients were classified into the β-blockers, the non-dihydropyridine calcium channel blocker (NDCCB), and the control groups according to the outpatient prescription within 2 weeks after hospital discharge.
Abbreviations: MACE, major adverse cardiac events;

| **Patients without receiving PCI/CABG**  **N= 4226** | **Weighted incidence rate (per 100 person-years)** | | | **Adjusted weighted rate ratio** | |
| --- | --- | --- | --- | --- | --- |
|  | β-blockers | NDCCB | Control | β-blockers vs. Control | β-blockers vs. NDCCB |
| 1-year mortality | 15.0 | 12.2 | 15.5 | 1.00(0.84-1.18) | 1.15(0.82-1.63) |
| Overall mortality | 10.3 | 9.9 | 11.4 | 0.94(0.85-1.04) | 1.01(0.84-1.21) |
| MACE in 1 year | 43.0 | 31.4 | 42.6 | 1.00(0.89-1.11) | 1.26(1.00-1.57)* |
| Repeated MI | 11.3 | 6.4 | 10.8 | 1.04(0.86-1.28) | 1.69(1.07-2.68)* |
| Repeated revascularization | 17.4 | 11.4 | 16.9 | 0.98(0.83-1.15) | 1.36(0.96-1.94) |
| Repeated ischemic stroke | 5.2 | 3.1 | 4.1 | 1.22(0.90-1.65) | 1.57(0.82-3.00) |

**p* <0.05, ***p* <0.01, ****p* <0.001.
